# Apparent Defect in Yeast Bud-Site Selection Due to a Specific Failure to Splice the Pre-mRNA of a Regulator of Cell-Type-Specific Transcription

**DOI:** 10.1371/journal.pone.0047621

**Published:** 2012-10-31

**Authors:** Shanshan Tuo, Kenichi Nakashima, John R. Pringle

**Affiliations:** Department of Genetics, Stanford University School of Medicine, Stanford, California, United States of America; Institut de Genetique et Microbiologie, France

## Abstract

The yeast *Saccharomyces cerevisiae* normally selects bud sites (and hence axes of cell polarization) in one of two distinct patterns, the axial pattern of haploid cells and the bipolar pattern of diploid cells. Although many of the proteins involved in bud-site selection are known, it is likely that others remain to be identified. Confirming a previous report (Ni and Snyder, 2001, Mol. Biol. Cell *12,* 2147–2170), we found that diploids homozygous for deletions of *IST3/SNU17* or *BUD13* do not show normal bipolar budding. However, these abnormalities do not reflect defects in the apparatus of bipolar budding. Instead, the absence of Ist3 or Bud13 results in a specific defect in the splicing of the *MAT* ***a*** *1* pre-mRNA, which encodes a repressor that normally blocks expression of haploid-specific genes in diploid cells. When Mat**a**1 protein is lacking, Axl1, a haploid-specific protein critical for the choice between axial and bipolar budding, is expressed ectopically in diploid cells and disrupts bipolar budding. The involvement of Ist3 and Bud13 in pre-mRNA splicing is by now well known, but the degree of specificity shown here for *MAT* ***a*** *1* pre-mRNA, which has no obvious basis in the pre-mRNA structure, is rather surprising in view of current models for the functions of these proteins. Moreover, we found that deletion of *PML1,* whose product is thought to function together with Ist3 and Bud13 in a three-protein retention-and-splicing (RES) complex, had no detectable effect on the splicing *in vivo* of either *MAT* ***a*** *1* or four other pre-mRNAs.

## Introduction

A central feature of cell polarization is selection of the appropriate axis. In the yeast *Saccharomyces cerevisiae*, this axis is defined by selection of the bud site, which occurs in one of two distinct patterns, depending on the cell type [1], [2]. In the axial pattern, as seen in Mat**a** or Matα cells (such as normal haploids), the first bud on a daughter cell is directly adjacent to its birth scar, and each subsequent bud site is adjacent to the immediately preceding division site; thus, the bud scars form a chain that begins at the birth-scar-proximal cell pole. In the bipolar pattern, as seen in Mat**a**/Matα cells (such as normal diploids), the first bud can be at either pole but is usually at the birth-scar-distal pole; subsequent buds can be at either pole, so that older cells typically show a cluster of bud scars around each cell pole. The axial pattern depends on a transient cortical marker that involves the proteins Axl1, Axl2/Bud10, Bud3, and Bud4 [2]–[10]. In contrast, the bipolar pattern depends on persistent cortical markers at both the birth-scar-proximal and distal cell poles that involve the proteins Bud8, Bud9, Rax1, and Rax2 [2], [10]–[16]. In both budding patterns, the positions of the cortical markers appear to be transmitted through a signal module based on the Ras-like protein Rsr1/Bud1 to the Rho-like protein Cdc42, whose localized activation then triggers the polarization of the cytoskeletal and secretary systems [10], [17], [18].

Of all the proteins known to be involved in determining the cell-type-specific budding patterns, only one is expressed in a cell-type-specific manner. Like other haploid-specific genes, *AXL1* is transcriptionally repressed in diploids by the Mat**a**1-Matα2 heterodimeric repressor [4], [19], [20]. When *AXL1* is ectopically expressed in diploid cells by expression from a heterologous promoter, the axial budding system is largely epistatic to the bipolar budding system, and many axial-like chains of bud scars are observed [4] (this study). Despite the importance of Axl1 for axial budding, cells lacking this protein (such as wild-type **a**/α diploid cells or *axl1* mutant haploid cells) show inefficient axial-like budding if the bipolar budding system is disabled by mutation of *RAX1*, *RAX2*, or both *BUD8* and *BUD9* [12], [14], [16], [20].

The original screens for bud-site-selection mutants did not appear to have been saturated, suggesting that there might be additional, unidentified proteins involved in either or both of the two budding patterns. Consistent with this possibility, Ni and Snyder [21] reported that a genomic-scale screen of diploid deletion mutants identified many additional genes that were necessary for normal bipolar budding. In re-testing these genes using freshly made deletions in our own strain background, we confirmed that deletion of either *BUD13* or *IST3*/*SNU17* had a strong effect on the budding pattern of diploid cells. Thus, we began to study these genes further in the initial hope that they would define additional proteins involved in bipolar budding. However, evidence soon began to accumulate that Bud13 and Ist3 function instead in pre-mRNA splicing [22]–[29]. This initially suggested that a protein with a direct role in bipolar budding was encoded by an intron-containing gene whose splicing required Bud13 and Ist3. Instead, however, we have shown that the budding-pattern phenotypes of *bud13Δ* and *ist3Δ* mutants are due to their highly inefficient splicing of *MAT* ***a*** *1* pre-mRNA; the resulting deficiency of Mat**a**1 protein allows expression of Axl1, with consequent disruption of the bipolar budding pattern. The highly specific effects of *bud13Δ* and *ist3Δ* mutations on *MAT* ***a*** *1* splicing and the failure of *pml1Δ* mutations to show similar effects raise questions for the current model that Bud13, Ist3, and Pml1 function together in a three-protein complex with a general role in pre-mRNA splicing and nuclear retention.

## Materials and Methods

### Strains, plasmids, genetic methods, and growth conditions

The strains and plasmids used in this study are described in Tables 1 and 2 and/or in the text below. Standard genetic and recombinant-DNA methods were used except where noted [30]–[32]. The polymerase chain reaction (PCR) used either the Expand High Fidelity System (Roche Molecular Biochemicals) or the PrimeSTAR system (Takara Bio) under standard conditions. The primers used are described in Table S1.

**Table 1:** *S. cerevisiae* strains used in this study.

Strain	Genotype^a^	Source
YEF473	**a**/α *his3-Δ200/his3-Δ200 leu2-Δ1/leu2-Δ1 lys2-801/lys2-801 trp1-Δ63/trp1-Δ63 ura3-52/ura3-52*	[49]
YEF473A	**a** *his3-Δ200 leu2-Δ1 lys2-801 trp1-Δ63 ura3-52*	Segregant from YEF473
YEF473B	α *his3-Δ200 leu2-Δ1 lys2-801 trp1-Δ63 ura3-52*	Segregant from YEF473
AM201	**a** *axl1Δ::HIS3*	This laboratory^b^
AM273	**a** *axl2Δ::HIS3*	This laboratory^b^
DDY210	**a**/α *bud3Δ::HIS3/bud3Δ::HIS3*	This laboratory^b^
KNY388	**a**/α *bud8Δ::TRP1/bud8Δ::TRP1 bud9Δ::His3MX6/bud9Δ::His3MX6*	This laboratory^b^
ML130	**a** *bar1Δ*	[14]
STY216	**a**/α *bud13Δ::His3MX6/BUD13*	See text
STY229	**a**/α *ist3Δ::His3MX6/IST3*	See text
STY237	α *bud13Δ::His3MX6*	Segregant from STY216
STY241	α *ist3Δ::His3MX6*	Segregant from STY229
STY254	**a**/α *bud13Δ::His3MX6/bud13Δ::His3MX6*	This study^c^
STY260	**a**/α *ist3Δ::His3MX6/ist3Δ::His3MX6*	This study^c^
STY450	**a**/α *pml1Δ::His3MX6/PML1*	See text
STY459	**a**/α *axl2Δ::HIS3/axl2Δ::HIS3 ist3Δ::His3MX6/ist3Δ::His3MX6*	This study^d^
STY460	**a** *pml1Δ::His3MX6*	Segregant from STY450
STY464	**a**/α *pml1Δ::His3MX6/pml1Δ::His3MX6*	This study^c^
STY484	**a**/α *axl1::HIS3/axl1::HIS3 bud13Δ::His3MX6/bud13Δ::His3MX6*	This study^e^
STY506	**a**/α *bud3Δ::HIS3/bud3Δ::HIS3 bud13Δ::TRP1/BUD13*	See text
STY550	**a**/α *bud3Δ::HIS3/bud3Δ::HIS3 bud13Δ::TRP1/bud13Δ::TRP1*	This study^f^
STY604	**a** *AXL1-GFP:TRP1*	See text
STY605	α *AXL1-GFP:TRP1*	Segregant from STY241×STY604
STY610	**a**/α *ist3Δ::His3MX6/ist3Δ::His3MX6 AXL1-GFP:TRP1/AXL1-GFP:TRP1*	This study^g^
STY611	**a**/α *bud13Δ::His3MX6/bud13Δ::His3MX6 AXL1-GFP:TRP1/AXL1-GFP:TRP1*	This study^h^
STY612	**a**/α *AXL1-GFP:TRP1/AXL1-GFP:TRP1*	STY604×STY605
STY619	**a**/α *pml1Δ::His3MX6/pml1Δ::His3MX6 AXL1-GFP:TRP1/AXL1-GFP:TRP1*	This study^i^
STY627	**a**/α *P_GAL1_-AXL1:His3MX6/AXL1*	See text
STY741	**a**/α *bud13Δ::His3MX6/bud13Δ::His3MX6* [*YCp111-P_ADH1_-MATa1*]	This study^j^
STY742	**a**/α *ist3Δ::His3MX6/ist3Δ::His3MX6* [*YCp111-P_ADH1_-MATa1*]	This study^j^

a All strains are congenic to YEF473 except as indicated.

b The mutations were generated by the PCR method [34], [35]. Each mutation is a complete replacement of the indicated ORF by the indicated selectable marker (A. McKenzie III, D. DeMarini, K. Nakashima, and J. R. Pringle, unpublished results).

c Constructed by mating STY237, STY241, or STY460 to an appropriate segregant from STY216, STY229, or STY450.

d Constructed by mating appropriate segregants from AM273×STY241.

e Constructed by mating appropriate segregants from AM201×STY237.

f Constructed by mating appropriate segregants from STY506.

g Constructed by mating appropriate segregants from STY241×STY604.

h Constructed by mating appropriate segregants from STY237×STY604.

i Constructed by mating appropriate segregants from STY460×STY605.

j Constructed by transforming plasmid YCp111-P_ADH1_-MATa1 into strains STY254 and STY260, respectively.

**Table 2:** Plasmids used in this study.

Plasmid	Description	Source
YEplac181	*LEU2* (high copy)	[50]
YEplac195	*URA3* (high copy)	[50]
YCplac111	*LEU2* (low copy)	[50]
YEpGFP*-BUD8F	*GFP-BUD8* a in YEplac181	[15]
YEpGFP*-BUD9	*GFP-BUD9* a in YEplac195	[15]
RAX2-GFP	*URA3 RAX2-GFP* (low copy)	A. Fujita
YCp111-P_ADH1_	*ADH1* promoter in YCp111	This study
YCp111-P_ADH1_-MATa1	*MAT* ***a*** *1* cDNA under *ADH1* promoter	See text

a The *GFP* allele encodes GFP with the F64L, S65T, and V163A substitutions.

Except where noted, cells were grown at 24°C on YM-P rich liquid medium, YPD rich solid medium, or synthetic complete (SC) medium lacking appropriate nutrients as needed to maintain plasmids or select transformants [30], [33]. All media contained 2% glucose except where noted. Cells expressing Green Fluorescent Protein (GFP)-tagged proteins were grown in the dark to minimize photobleaching.

To construct strains in which the complete *BUD13*, *IST3*, or *PML1* open reading frame (ORF) was deleted, the PCR method [34] was used with plasmid pFA6a-His3MX6 or pFA6a-TRP1 [35] as template. The *His3MX6*-containing cassettes were transformed into strain YEF473, and the *bud13Δ::TRP1* cassette was transformed into strain DDY210. Proper integration of the cassettes was confirmed both by PCR checks [35] and by verifying 2∶2 segregation of the selectable marker. The PCR method was also used to construct a strain expressing *AXL1* under control of the *GAL1* promoter and a strain in which the chromosomal *AXL1* locus was tagged with *GFP* sequences at its C-terminus, using plasmids pFA6a-His3MX6-PGAL1 and pFA6a-GFP(S65T)-TRP1 [35] as templates. The PCR products were transformed into strains YEF473 and YEF473A, respectively. Proper integration of the cassettes was confirmed by observing 2∶2 segregation of the selectable marker and the presence of the expected *AXL1-*related phenotypes.

To clone a *MAT* ***a*** *1* cDNA under control of the *ADH1* promoter, the 414 bp upstream of the *ADH1* ORF were amplified using genomic DNA from strain YEF473 as template and the primers described in Table S1. The product was digested with *Sph*I (site in the chromosomal sequence) and *Sal*I (site included in the primer) and cloned into plasmid YCplac111 using the corresponding sites to produce plasmid YCp111-P_ADH1_. The *MAT* ***a*** *1* cDNA amplified by reverse-transcription-PCR (RT-PCR) from wild-type cells was digested with *Sal*I and *Bam*HI (sites included in the *MAT* ***a*** *1* primers; Table S1) and cloned into YCp111-P_ADH1_ using the corresponding sites to yield plasmid YCp111-P_ADH1_-MATa1, whose structure was confirmed by DNA sequencing.

### RT-PCR of mRNA and pre-mRNA

To examine the splicing of *MAT* ***a*** *1* and other pre-mRNAs, total RNA was prepared from the strains of interest by the hot-phenol method [36]. The RNA was then treated with the DNA-*free*™ DNase-treatment-and-removal kit (Applied Biosystems) as recommended by the manufacturer, and RT-PCR was conducted in a two-step reaction. Single-stranded cDNA was synthesized using an oligo (dT)_16_ primer, MultiScribe™ reverse transcriptase (Applied Biosystems), and a regimen of 10 min at 25°C, 30 min at 48°C, and 5 min at 95°C, followed by storage at 4°C. cDNAs were then amplified by PCR using PrimeSTAR polymerase (Takara Bio) and gene-specific primers (Table S1). PCR was conducted using a regimen of 4 min at 94°C; 30 cycles of 10 s at 98°C, 5 s at 55°C, and 45 s (*MAT* ***a*** *1*), 60 s (*ACT1* and *RPS17A*), or 90 s (*DYN2* and *RPL7A*) at 72°C; 10 min at 72°C; and storage at 4°C. The products were then separated on a 3% agarose gel, stained with ethidium bromide, and imaged using an AlphaImager and AlphaEase®FC software (Alpha Innotech). The 1-kb DNA ladder was purchased from Invitrogen.

### Staining and microscopy

To visualize bud scars and birth scars, cells were grown to exponential phase, stained with 200 µg/ml Calcofluor [11], and examined using a Nikon Eclipse 600 FN microscope equipped with a Hamamatsu ORCA-2 CCD camera and an Apo 100×/1.40 NA oil-immersion objective. To visualize GFP-fusion proteins, cells were grown to exponential phase and observed using the same microscope. All images were collected using MetaMorph software (Molecular Devices). Images of GFP-tagged proteins were taken with an exposure time of 3 s, and exposure times for Calcofluor images were ∼30 ms.

### Halo assay for α-factor production

The strains to be tested were cultured in YM-P medium at 30°C overnight until stationary phase. The OD_600_ of a 10-fold dilution of each culture was checked, and 10-µl samples (containing 7 to 10 µl of the undiluted culture, as needed to achieve equal OD units, plus additional YM-P medium as needed) were spotted onto a YPD plate and grown for 4 days. A second YPD plate was spread with ∼10^7^ cells of the α-factor-supersensitive strain ML130, the patches of the strains to be tested were replicated onto this plate, and incubation was continued for 3 days before photographing the plate.

### Growth-rate measurements

To measure growth rates in liquid culture, the strains were grown overnight in SC medium (until OD_600_≈0.4–0.5) at 24°C, 30°C, and 37°C. Each culture was diluted two-fold with fresh SC medium pre-warmed to the same temperature, and doubling times were determined as the times required to return to the original OD_600_. The measurement was repeated three times for each strain and temperature.

## Results

**Figure 1:**
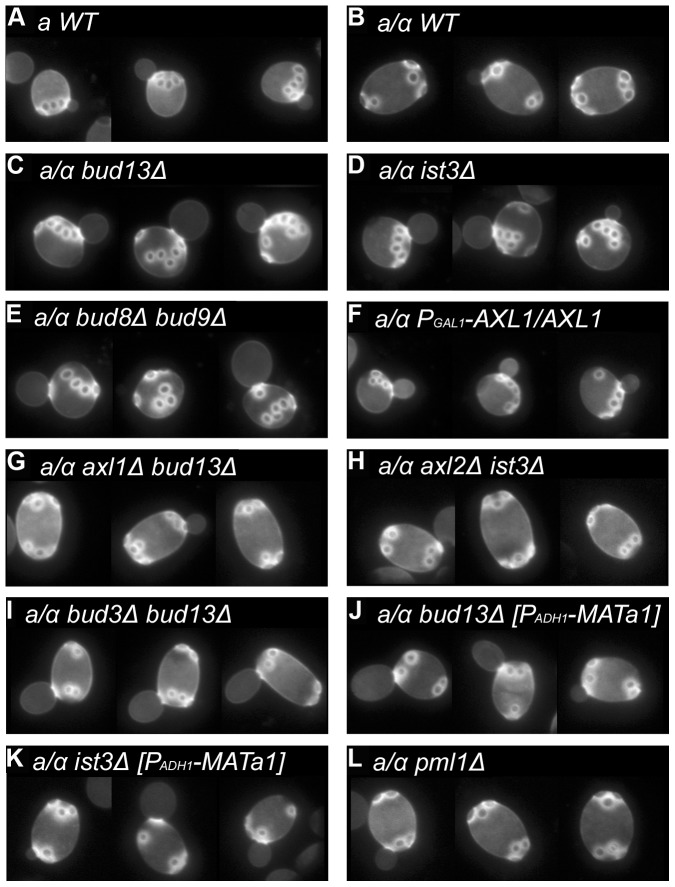
Budding-pattern phenotypes of wild-type and mutant strains. Strains with the indicated genotypes were grown to exponential phase in SC medium and stained with Calcofluor to visualize bud scars. A montage of representative cells is shown for each strain. Strains used were (A) YEF473A; (B) YEF473; (C) STY254; (D) STY260; (E) KNY388; (F) STY627 (grown on 2% galactose +2% raffinose instead of glucose and at 30°C instead of 22°C); (G) STY484; (H) STY459; (I) STY550; (J) STY741; (K) STY742; (L) STY464.

The major features of the normal axial and bipolar budding patterns (see Introduction) are illustrated in Figure 1A–B and Table 3, lines 1 and 2. As reported by Ni and Snyder [21], we found that deletion of *IST3* or *BUD13* had no detectable effect on the axial budding pattern of haploid cells (data not shown) but profoundly affected the bipolar budding pattern of homozygous diploid mutants. In the mutants, use of the birth-scar-distal pole for the first bud on daughter cells was largely, although not entirely, lost (Table 3, lines 3 and 4), and cells that had budded multiple times showed many scars that were not at either pole; these scars were often present in chains reminiscent of those in axially budding cells (Figure 1C–D).

**Table 3:** Positions of first bud sites on daughter cells of wild-type and mutant strains.^a^

Line	Genotype	Position of first bud site		
		Distal	Equatorial	Proximal
1	***a*** * WT*	0	0	120
2	***a*** */α WT*	130	0	0
3	***a*** */α bud13Δ*	14	0	142
4	***a*** */α ist3Δ*	49	0	102
5	***a*** */α bud8Δ bud9Δ*	7	20	134
6	***a*** */α P_GAL1_-AXL1/AXL1*	33	0	146
7	***a*** */α axl1Δ bud13Δ*	100	0	0
8	***a*** */α axl2Δ ist3Δ*	122	1	0
9	***a*** */α bud3Δ bud13Δ*	100	1	0
10	***a*** */α bud13Δ* [*P_ADH1_-MATa1*]	139	0	30
11	***a*** */α ist3Δ* [*P_ADH1_-MATa1*]	142	0	9
12	***a*** */α pml1Δ*	131	0	0

a The strains and culture conditions used were the same as in Figure 1. The numbers of daughter cells that produced first buds near the birth-scar-distal pole, the birth-scar-proximal pole, or neither pole (equatorial region) were counted.

**Figure 2:**
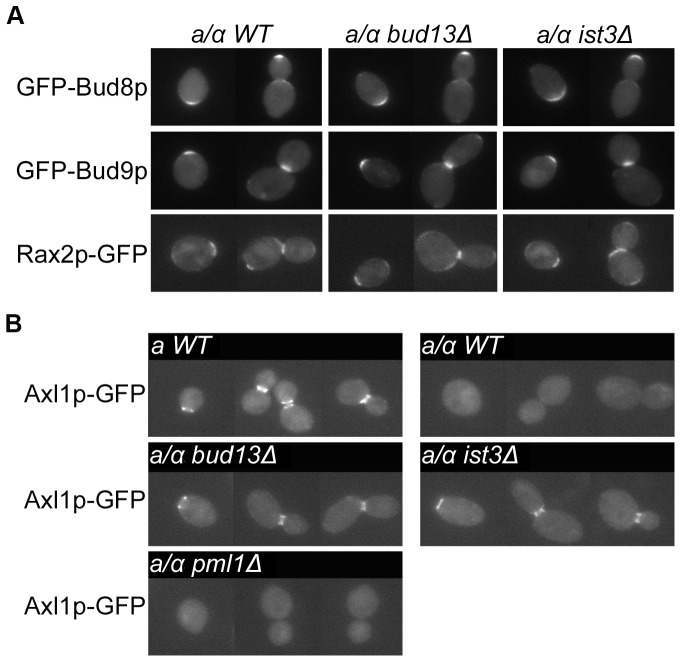
Expression and localization of cortical marker proteins in wild-type and mutant strains. In each panel, a montage of representative cells is shown. (A) Localization of the bipolar marker proteins Bud8, Bud9, and Rax2. Wild-type diploid strain YEF473, *bud13Δ/bud13Δ* strain STY254, and *ist3Δ/ist3Δ* strain STY260 were transformed with plasmid YEpGFP*-BUD8F, YEpGFP*-BUD9, or RAX2-GFP, and cells grown to exponential-phase in SC-Leu or SC-Ura medium were examined for the localization and intensity of GFP fluorescence. (B) Expression and localization of the haploid-specific axial-marker protein Axl1. Wild-type *MAT* ***a*** strain STY604, wild-type *MAT* ***a*** */MATα* strain STY612, and *MAT* ***a*** */MATα* strains homozygous for *bud13Δ* (STY611), *ist3Δ* (STY610), or *pml1Δ* (STY619), each of which expresses *AXL1-GFP* from the chromosomal *AXL1* locus, were grown to exponential phase in SC medium and examined for the expression and localization of GFP fluorescence. Exposure time and scaling factor were identical for each image in panel B.

This phenotype resembled the inefficient axial budding seen in diploid cells when bipolar budding is disabled by mutation of *RAX1, RAX2,* or both *BUD8* and *BUD9* (see Introduction; Table 3, line 5; Figure 1E), suggesting that the *ist3* and *bud13* mutants might be generally defective in generating the signals for bipolar budding. However, examination of the localizations of Bud8, Bud9, and Rax2 in the mutants revealed patterns indistinguishable from those seen in wild-type cells (Figure 2A). These observations suggested the alternative hypothesis that the *ist3* and *bud13* mutants might have a partially functional axial-budding system in diploid cells, such as what occurs when Axl1 is ectopically expressed in such cells (see Introduction; Table 3, line 6; Figure 1F). In support of this possibility, *ist3* and *bud13* mutant diploids showed normal bipolar budding when a gene important for axial budding was also deleted (Table 3, lines 7–9; Figure 1G–I). Moreover, when the chromosomal *AXL1* gene was tagged at its 3′ end with *GFP*, the Axl1-GFP fusion protein was not detectable in wild-type diploid cells (as expected), but it was present in its normal (for haploid cells) localization at the mother-bud neck and division site in *ist3* and *bud13* mutant diploid cells, although its levels appeared lower than those in wild-type haploid cells (Figure 2B, top four panels).

**Figure 3:**
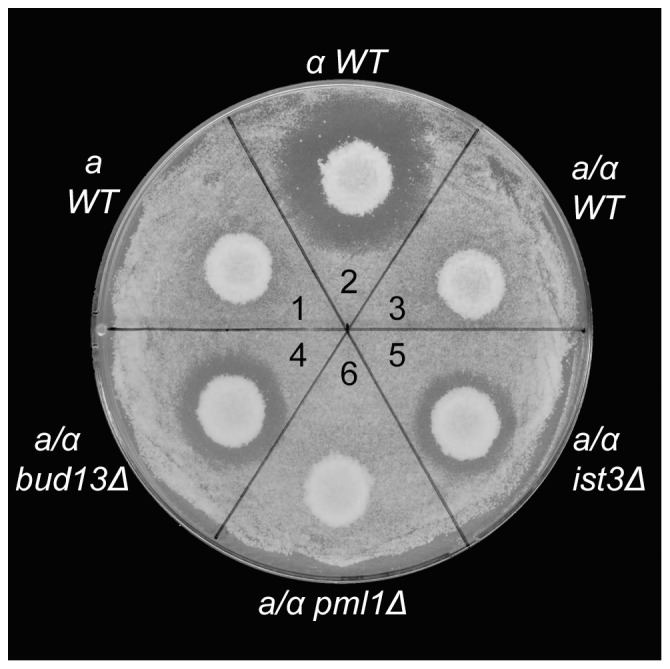
α-factor production by *bud13Δ* and *ist3Δ* diploid strains. Colonies of wild-type (YEF473A, *MAT* ***a***; YEF473B, *MATα*; and YEF473, *MAT* ***a*** */MATα*) and mutant (STY254, *bud13Δ/bud13Δ*; STY260, *ist3Δ/ist3Δ*; and STY464, *pml1Δ/pml1Δ*) strains were replicated into a lawn of strain ML130 (*MAT* ***a*** *bar1Δ*) on a YPD plate (see Materials and Methods). α-factor production results in a halo of growth inhibition.

The accumulating evidence that Ist3 and Bud13 are involved in pre-mRNA splicing (see Introduction) suggested that these proteins might be particularly important for splicing the pre-mRNA of *MAT* ***a*** *1*. This gene contains two introns [37] and encodes one subunit of a heterodimeric repressor that normally prevents the expression of haploid-specific genes (such as *AXL1*) in diploid cells [19]. In this case, the expression of other haploid-specific genes should also be derepressed by a lack of Mat**a**1 in *ist3* and *bud13* mutants. Indeed, a halo assay showed that the mating pheromone α-factor, which is normally expressed only in Matα haploid cells (Figure 3, sectors 1–3), was also expressed in *ist3* and *bud13* mutant diploid cells (Figure 3, sectors 4 and 5). The levels of α-factor secreted by the mutants appeared to be somewhat lower than those from a wild-type Matα strain, suggesting that there might be some residual Mat**a**1 in the mutant cells.

**Figure 4:**
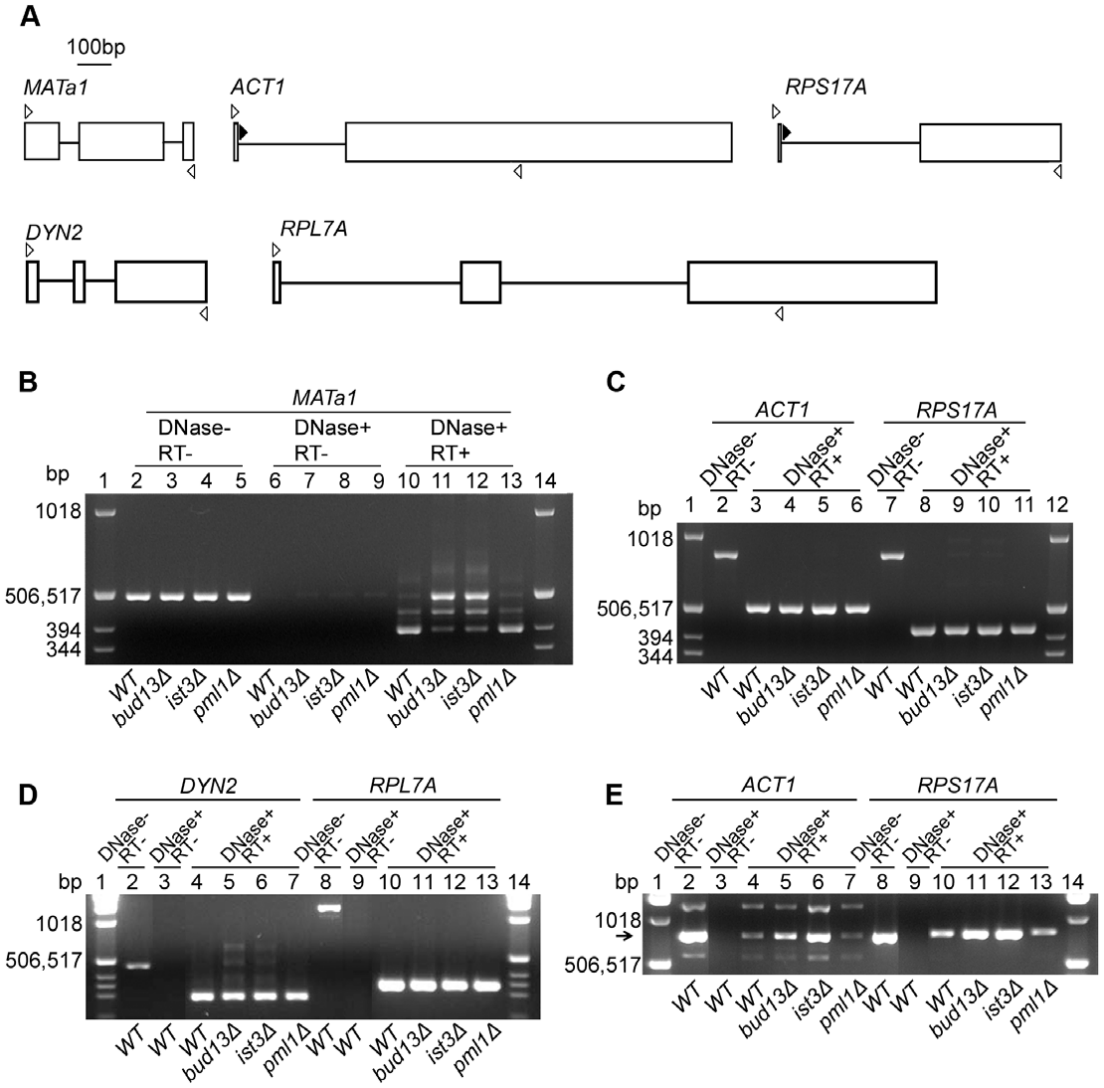
Inefficient splicing of *MATa1* pre-mRNA, but not of other pre-mRNAs, in *bud13Δ* and *ist3Δ* mutants. (A) Schematic representations of the genes tested and the primers used. Exons and introns are shown as open boxes and lines, respectively. Open arrowheads, forward and reverse primers corresponding to exon sequences; closed arrowheads, alternative forward primers corresponding to *ACT1* and *RPS17A* intron sequences (see Table S1). (B–D) Analyses of spliced and unspliced mRNAs using the exon-derived primers. Cells of diploid strains YEF473 (wild-type), STY254 (*bud13Δ/bud13Δ*), STY260 (*ist3Δ/ist3Δ*), and STY464 (*pml1Δ/pml1Δ*) were grown in YM-P medium at 30°C to OD_600_≈0.5. Total RNA was prepared, treated with DNase, and reverse-transcribed into single-stranded cDNA using oligo (dT)_16_ primers (see Materials and Methods). cDNAs were then amplified by PCR using the appropriate primers. RNA samples that were not treated with DNase (DNase-) and/or not subjected to reverse transcription (RT−), as indicated, were used as controls. Molecular-size markers were run in the outside lanes in each gel; their sizes are indicated. (E) Analysis of unspliced *ACT1* and *RPS17A* transcripts using the intron-derived forward primers. The reverse primers and other conditions were as described for B–D. The arrow indicates the expected size for the cDNA derived from unspliced pre-mRNA (approximately the same for each gene). The other bands in the *ACT1* lanes appear to be nonspecific PCR products.

To ask directly if Ist3 and Bud13 are involved in the splicing of *MAT* ***a*** *1* pre-mRNA, we used RT-PCR to examine the levels of the pre-mRNA and its splice products in various strains. In wild-type cells, most *MAT* ***a*** *1* RNA was present as the fully spliced product (Figure 4B, lane 10), although a significant amount of partially spliced product was also detected, reflecting differentially efficient splicing of the two introns, as reported previously [37]–[40]. In contrast, although fully spliced product was detected in the *ist3* and *bud13* mutant cells, its amount was greatly reduced relative to the partially spliced and unspliced RNAs (Figure 4B, lanes 11 and 12; note co-migration of the band derived from unspliced pre-mRNA with the band generated from contaminating DNA as PCR template when DNase treatment was omitted: Figure 4B, lanes 2–5). If the *ist3* and *bud13* mutant budding-pattern phenotypes reflect a reduced level of Mat**a**1 protein due to inefficient splicing of *MAT* ***a*** *1* pre-mRNA, then expression of an intron-free *MAT* ***a*** *1* cDNA under control of a constitutive promoter should suppress the mutant phenotypes, and this was indeed the case (Table 3, lines 10 and 11; Figure 1J–K).

To ask if the effects of *ist3* and *bud13* mutations are specific to *MAT* ***a*** *1* pre-mRNA, we also examined the splicing of the *ACT1, RPS17A, DYN2,* and *RPL7A* pre-mRNAs; the latter two genes were chosen specifically because, like *MAT* ***a*** *1,* they contain two introns. Using primers derived from exon sequences and thus capable of amplifying both spliced and unspliced mRNAs (Figure 4A, open arrowheads), we saw little or no effect of the mutations on pre-mRNA processing for any of these four genes (Figure 4C, lanes 2–5 and 7–10; Figure 4D, lanes 2–6 and 8–12), in striking contrast to *MAT* ***a*** *1*. For *ACT1* and *RPS17A,* we also examined the products obtained using forward primers derived from intron sequences (such that only the unspliced pre-mRNAs could be amplified: Figure 4A, closed arrowheads). In each case, we saw a modest but significant increase in the amount of pre-mRNA-derived product (Figure 4E, lanes 2–6 and 8–12). Taken together, these data suggest that Ist3 and Bud13 play a major role in the splicing of *MAT* ***a*** *1* pre-mRNA but only a minor role in the splicing of many other pre-mRNAs that contain either one or two introns.

**Figure 5:**
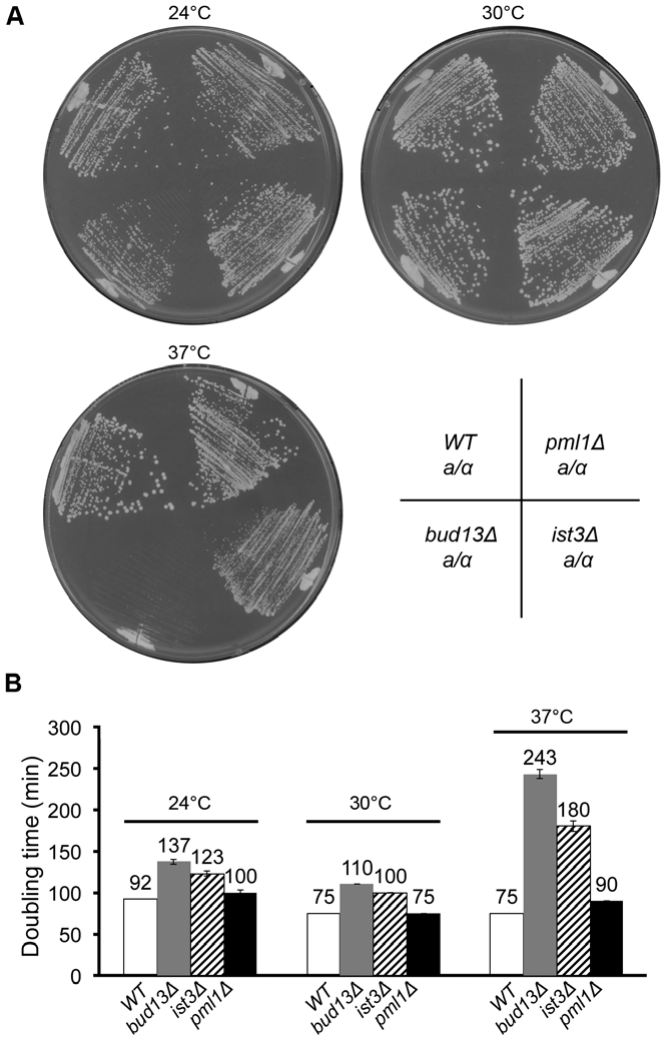
Growth rates of wild-type and mutant strains. Strains YEF473 (wild type), STY254 (*bud13Δ/bud13Δ*), STY260 (*ist3Δ/ist3Δ*), and STY464 (*pml1Δ/pml1Δ*) were tested for growth on both solid (A) and liquid (B) SC medium at the indicated temperatures. Doubling times in liquid culture were determined as described in Materials and Methods. Mean doubling times and standard deviations are indicated.

If Ist3 and Bud13 played a major role in the splicing of pre-mRNAs other than those we tested, it would be likely that at least one of the affected genes would be important for vegetative growth, so that the *ist3* and *bud13* mutants would show a significant growth defect relative to wild type. However, we observed only modest effects of the mutations on growth rates on either solid or liquid medium at 24°C or 30°C (Figure 5A–B). In contrast, a much larger effect was seen at 37°C (Figure 5A–B); although other explanations for this difference are possible, one plausible interpretation is that Ist3 and Bud13 may play a more important general role in pre-mRNA splicing at higher growth temperatures.

Strong evidence now suggests that Ist3 and Bud13 function, at least in part, in a retention-and-splicing (RES) complex with a third protein, Pml1 [26], [28], [41]–[43]. However, we could observe no effect of a *pml1* mutation on bipolar budding (Table 3, line 12; Figure 1L), expression of Axl1p or α-factor (Figure 2B, bottom panel; Figure 3, sector 6), the splicing of any of the five pre-mRNAs tested (Figure 4B, lane 13; Figure 4C, lanes 6 and 11; Figure 4D, lanes 7 and 13; Figure 4E, lanes 7 and 13), or growth rate at any of the three temperatures tested (Figure 5A–B). These data suggest that at least in our strain background, Pml1 either does not play a major role or is functionally redundant for its role in the function of Ist3 and Bud13 in the splicing of either *MAT* ***a*** *1* or other pre-mRNAs.

## Discussion

We re-tested many of the genes reported by Ni and Snyder [21] to be involved in bipolar budding. Among the most promising were *IST3* and *BUD13*: homozygous diploid *ist3* and *bud13* deletion strains had strong phenotypes that suggested a general impairment of bipolar budding. However, we have shown here that the budding-pattern phenotypes of these mutants reflect the roles of Ist3 and Bud13 in the splicing of the pre-mRNA for a cell-type-regulatory protein rather than any direct involvement in the mechanisms of bud-site selection.

### Roles of Ist3, Bud13, and Pml1 in pre-mRNA splicing

A role for Ist3/Snu17 in pre-mRNA splicing was first suggested by Gottschalk *et al.* [22], who reported that it was part of the U2 snRNP and the spliceosome and that its loss resulted in a general splicing defect and consequent slow growth. Studies by Rymond and co-workers further supported a role of Ist3 in the SF3b subcomplex of the U2 snRNP [25], [28] and also provided both genetic and biochemical data suggesting that Bud13 is also present in SF3b [24], [28]. Pml1 was not observed to be associated with SF3b but was found to co-purify with Ist3 and Bud13, consistent with the presence of a separate complex, as was indeed reported by Dziembowski *et al.* [26]. The latter workers did not find Ist3 in SF3b but instead only in a trimeric complex with Bud13 and Pml1; they named this complex “RES” for its apparent roles in nuclear retention and splicing. Subsequent studies have provided detailed information about the structure of this complex [41]–[43], but understanding of its function has lagged behind.

Both the studies cited above and early [23] and more recent [44] global analyses using microarrays have suggested that Ist3, Bud13, and Pml1 play a general role in pre-mRNA nuclear retention and splicing. However, such a general role appears difficult to reconcile with the modest growth defects observed for *ist3, bud13,* and *pml1* mutants at temperatures from 24–30°C [22], [26] (Figure 5). Moreover, studies by Spingola and co-workers have suggested that the roles of Ist3 and Bud13 might be more restricted; in particular, they found that the sporulation-specific splicing factor Mer1 is involved in the splicing of particular introns that contain a “Mer1-enhancer element”, that Ist3 is essential for Mer1 function, and that Bud13 is critical for the action of Mer1 on a subset of the introns whose splicing it activates [27], [29]. In addition, Schmidlin *et al.* [40] observed that an *ist3* mutation differentially affected the splicing of *MAT* ***a*** *1* pre-mRNA relative to that of the essential gene *ACT1* (and, presumably, other essential genes), to the point that a homozygous *ist3Δ* diploid strain grew well but could mate like a Matα haploid because of the lack of Mat**a**1 repressor.

Our own results suggest that Ist3 and Bud13 may play major roles in the splicing of only a minority of pre-mRNAs, at least at lower growth temperatures. RT-PCR using exon-derived primers showed a strong effect of *ist3* and *bud13* mutations on the splicing of *MAT* ***a*** *1* pre-mRNA *in vivo* (Figure 4B) but little or no effect on the splicing *in vivo* of any of the other four genes tested (Figure 4C–D), although a more sensitive test of RT-PCR using an intron-derived primer revealed modest but significant effects of these mutations on the splicing *in vivo* of the two genes tested (Figure 4E). Consistent with these data, the *ist3Δ* and *bud13Δ* mutations had only modest effects on growth rates at 24 or 30°C. The effects on growth rates were more substantial at 37°C, suggesting that Ist3 and Bud13 might be more generally important for splicing at higher growth temperatures.

Our results are also somewhat difficult to reconcile with the model that Ist3, Bud13, and Pml1 function together in splicing as a three-protein complex. If this were so, the corresponding deletion mutants would be expected to have similar phenotypes. However, in none of our assays (even those using the intron-derived primers) did we see any effect of a *pml1Δ* mutation that paralleled the effects of the *ist3Δ* and *bud13Δ* mutations. Moreover, the *pml1Δ* mutation showed very little effect on growth rate at any of the temperatures tested (Figure 5). Previous investigators have also observed only modest effects of *pml1* mutations on splicing (26,42), but these observations remain unexplained and might possibly reflect functional redundancy of Pml1 with an as yet unidentified protein.

Differential effects on the splicing of *MAT* ***a*** *1* relative to other pre-mRNAs have been observed previously. Nakazawa *et al.* [39] found that an ethyl-methane-sulfonate-induced mutation (presumably a hypomorphic point mutation) in the essential splicing factor Aar2 [45] affected the splicing of *MAT* ***a*** *1* but not that of *ACT1.* In addition, as noted above, Schmidlin *et al.* [40] reported previously that an *ist3Δ* mutation affected the splicing of *MAT* ***a*** *1* much more than that of *ACT1.* (Their results with a third gene, *IST1,* are more difficult to interpret.)

Why might the splicing of *MAT* ***a*** *1* be differentially dependent on Ist3 and Bud13? First, a gene with atypical splice-junction and/or branchpoint sequences might require special alternative and/or accessory factors for splicing. However, both *MAT* ***a*** *1* introns have consensus sequences in all three positions [37], [46] (http://www.yeastgenome.org/cgi-bin/locus.fpl?locus=YCR097W). Second, the splicing of a pre-mRNA with two introns (of which there are only 10–15 known examples in yeast) might have special requirements for which Ist3 and Bud13 are particularly important. However, we saw little or no effect of *ist3* or *bud13* mutations on the splicing of two other pre-mRNAs with two introns (Figure 4D). Finally, it might be that Ist3 and Bud13 are particularly important for the splicing (and/or nuclear retention) of pre-mRNAs with particularly short introns, a possibility raised by the fact that the *MAT* ***a*** *1* introns are (at 54 and 52 nucleotides, respectively) the two shortest known introns in yeast [47] (http://intron.ucsc.edu/yeast4.1]. Although we cannot rule out this possibility, it does not seem likely given (i) that the two introns in *DYN2* (where little or no effect was observed: Figure 4D) are also relatively short (at 96 and 80 nucleotides, respectively) and (ii) that the yeast gene with the third-shortest intron (*TAD3,* which has two introns of 68 and 56 nucleotides, respectively) is essential [48], so that a significant defect in its splicing in *ist3* and *bud13* mutants would be expected to have a noticeable effect on growth rate.

In summary, our observations of the considerable specificity of Ist3 and Bud13 for the splicing of *MAT* ***a*** *1* pre-mRNA, and of the apparent lack of involvement of Pml1 in this process, pose challenges for the model that a trimeric RES complex of Ist3, Bud13, and Pml1 plays a general role in nuclear retention and splicing.

### Bipolar bud-site selection

Given the evidence that Ist3 and Bud13 are involved in pre-mRNA splicing, we initially hypothesized that identification of the splicing target relevant to bud-site selection would reveal a previously unidentified protein that was important for bipolar budding. Instead, however, our results show unequivocally that the effects of *ist3* and *bud13* mutations on bipolar budding are accounted for completely by their effects on the splicing of *MAT* ***a*** *1* pre-mRNA, with the resulting ectopic expression in diploid cells of the normally haploid-specific bud-site-selection protein Axl1. Why the selection of axial budding sites is epistatic to the selection of bipolar budding sites when all the factors needed for both patterns are present (as is the case in diploid cells expressing Axl1) remains one of the key unanswered questions about the mechanisms of bud-site selection. Also remaining open is the important question of whether proteins other than Bud8, Bud9, Rax1, and Rax2 are involved in marking the sites for bipolar budding.

## Supporting Information

Table S1 PCR primers used in this study.
(DOCX)
