# Supplementary material for: Apparent Defect in Yeast Bud-Site Selection Due to a Specific Failure to Splice the Pre-mRNA of a Regulator of Cell-Type-Specific Transcription
Source: PLoS One. 2012 Oct 31;7(10):e47621. doi: 10.1371/journal.pone.0047621 (PMC3485267; doi:10.1371/journal.pone.0047621)
Supplement: Table S1 — PCR primers used in this study. (DOCX) [file pone.0047621.s001.docx]

| **Table S1.** PCR primers used in this study. | |
| --- | --- |
| **Primer** | **Sequence** |
| F1/BUD13 ^a^ | 5'-AGACTCGAATGGTGGAAGATAACAACAGGACGTTTATTACCGGATCCCCGGGTTAATTA-3' |
| R1/BUD13 ^a^ | 5'-TACACAAAGCTTTCCGCATAGTTATATATTATCTCATTTGAATTCGAGCTCGTTTAAAC-3' |
| F/BUD13-chk ^a^ | 5'-TACGATAGTGAACCTCTGCTGATTC-3' |
| 3'TRP1-chk ^a^ | 5'-GTGCTTAATCACGTATACTCACGTGCTCAA-3' |
| F1/IST3 ^b^ | 5'-ATTCTAGATCAAGAACATAGATAATATAAACAAAATAACACGGATCCCCGGGTTAATTA-3' |
| R1/IST3 ^b^ | 5'-CTATATGAATATAAGATATGCGATGAAAGAAAAAATTATGAATTCGAGCTCGTTTAAAC-3' |
| F/IST3-chk ^b^ | 5'-CGCTTATCAGAAGAGCTGAAGCAAT-3' |
| F1/PML1 ^c^ | 5'-GCATGGTGTACTTCATTTCCGACTCCATTTGCGTATAGACCGGATCCCCGGGTTAATTAA-3' |
| R1/PML1 ^c^ | 5'-AATAATTAAAACACACTGAAAGTGTGTTTCTTATATATGGGAATTCGAGCTCGTTTAAAC-3' |
| F/PML1-chk ^c^ | 5'-GAGAACGGCTGTCCGAAACCAACGT-3' |
| R/PML1-chk ^c^ | 5'-CAAAGAATTTCAAAGGGCGCTATTA-3' |
| 3'PTEF-chk ^a,b,c^ | 5'-GTATGGGCTAAATGTACGGGCGACAGTCAC-3' |
| 5'TTEF-chk ^c^ | 5'-TATTTTTTTTCGCCTCGACATCATCTGCCC-3' |
| F/PGAL1-AXL1 ^d^ | 5'-CTGGCGTTAAAAAATAGCAACTGAATAAGTTTTTTTACTGGAATTCGAGCTCGTTTAAAC-3' |
| R/PGAL1-AXL1 ^d^ | 5'-AAAACGAGACTTCATAATTAGTTACTTCTCTCAAGGACATTTTGAGATCCGGGTTTT-3' |
| F/AXL1-GFP ^d^ | 5'-GGAGCTTCCTGAACCAAACTTTTTCCGCAAGGCCGCATTTCGGATCCCCGGGTTAATTAA-3' |
| R/AXL1-GFP ^d^ | 5'-CAAAAACGTGGAAAGGCTGGAACGAGCAAAATACGGTTCAGAATTCGAGCTCGTTTAAAC-3' |
| F/MATa1-SalI ^e^ | 5'-AAAGTCGACATGGATGATATTTGTAGTATGGCGG-3' |
| R/MATa1-BamHI ^e^ | 5'-GGGATCCCTTATTTAGATCTCATACGTTTATTT-3' |
| F/PADH1-SphI ^f^ | 5'-AAAGCATGCAACTTCTTTTCTTTTTTTT-3' ^d^ |
| R/PADH1-SalI ^f^ | 5'-AAAGTCGACCATTGTATATGAGATAGTTGATTGT-3' ^d^ |
| F/ACT1-15 ^g^ | 5'-TTTACTGAATTAACAATGGATTCTG-3' |
| R/ACT1+786 ^g^ | 5'-CAGCGTAAATTGGAACGACGTGAGT-3' |
| F/ACT1+11 ^h^ | 5'-GTATGTTCTAGCGCTTGCACCATCC-3' ^e^ |
| F/RPS17A-22 ^e^ | 5'-TCTCGAGACTAGCAATAACAAAATG-3' |
| R/RPS17A+785 ^e^ | 5'-TTAAACTCTCTTTCTGTAACGTCTG-3' |
| F/RPS17A+4 ^h^ | 5'-GTATGTTAATATGGACTAAAGGAGG-3' ^e^ |
| F/DYN2 ^e^ | 5'-ATGAGCGATGAAAATAAGAGTACGC-3'^f^ |
| R/DYN2 ^e^ | 5'-TTATGCTGTTTTGAAAACTAAAAAC-3' ^f^ |
| F/RPL7A-14 ^g^ | 5'-AAATTAAGATCACAATGGCCGCTGA-3' ^f^ |
| R/RPL7A+1232 ^g^ | 5'-TCTTGTCAATCTTAGCAATTGTAGA-3' ^f^ |

^a^ Primers used for deletion and checking of *BUD13.*

^b^ Primers used for deletion and checking of *IST3.*

^c^ Primers used for deletion and checking of *PML1.*

^d^ Primers used to construct the chromosomal *P_GAL1_-AXL1* and *AXL1-GFP* loci.

^e^ Primers designed to amplify the full-length coding regions (and hence cDNAs derived from either spliced or unspliced mRNAs) of *MAT****a****1,* *RPS17A,* and *DYN2.* See Figure 4A.

^f^ Primers used to clone the *ADH1* promoter.

^g^ Primers corresponding to exon sequences (the forward primers include the start codons) and designed to amplify segments of *ACT1* and *RPL7A* that include their introns (and hence cDNAs derived from either spliced or unspliced mRNAs). See Figure 4A.

^h^ Forward primers corresponding to intron sequences that should amplify only cDNAs derived from the unspliced pre-mRNAs. See Figure 4A.
